# Supplementary material for: HIVprotI: an integrated web based platform for prediction and design of HIV proteins inhibitors
Source: J Cheminform. 2018 Mar 9;10:12. doi: 10.1186/s13321-018-0266-y (PMC5845081; doi:10.1186/s13321-018-0266-y)
Supplement: Supplementary file 2 — Additional file 2. Source code of HIVProtI web server. [file 13321_2018_266_MOESM2_ESM.zip › HIVprotI_Source-code/hivprotI/weka-3-6-6/doc/weka/filters/unsupervised/attribute/RemoveType.html]

RemoveType


---


|  |  |  |
| --- | --- | --- |
| **Overview** \| **Package** \| **Class** \| **Tree** \| **Deprecated** \| **Index** \| **Help** \| **Weka's home** | |  |
| **PREV CLASS**   **NEXT CLASS** | **FRAMES**    **NO FRAMES**     **All Classes** |  |
| SUMMARY: NESTED \| FIELD \| CONSTR \| METHOD | DETAIL: FIELD \| CONSTR \| METHOD |  |


---


## weka.filters.unsupervised.attribute Class RemoveType

```
java.lang.Object
  weka.filters.Filter
      weka.filters.unsupervised.attribute.RemoveType
```

**All Implemented Interfaces:**: java.io.Serializable, CapabilitiesHandler, OptionHandler, RevisionHandler, StreamableFilter, UnsupervisedFilter

---

``` public class RemoveType extends Filter implements UnsupervisedFilter, StreamableFilter, OptionHandler ```

Removes attributes of a given type.


Valid options are:

```
 -T <nominal|numeric|string|date|relational>
  Attribute type to delete. Valid options are "nominal", 
  "numeric", "string", "date" and "relational".
  (default "string")
```

```
 -V
  Invert matching sense (i.e. only keep specified columns)
```

**Version:**
:   $Revision: 5547 $

**Author:**
:   Richard Kirkby (rkirkby@cs.waikato.ac.nz)

**See Also:**: Serialized Form

---

| **Field Summary** | |
| --- | --- |
| `static Tag[]` | `TAGS_ATTRIBUTETYPE`             Tag allowing selection of attribute type to delete |


**Constructor Summary**

`RemoveType()`


| **Method Summary** | |
| --- | --- |
| `java.lang.String` | `attributeTypeTipText()`             Returns the tip text for this property |
| `boolean` | `batchFinished()`             Signify that this batch of input to the filter is finished. |
| `SelectedTag` | `getAttributeType()`             Gets the attribute type to be deleted by the filter. |
| `Capabilities` | `getCapabilities()`             Returns the Capabilities of this filter. |
| `boolean` | `getInvertSelection()`             Get whether the supplied columns are to be removed or kept |
| `java.lang.String[]` | `getOptions()`             Gets the current settings of the filter. |
| `java.lang.String` | `getRevision()`             Returns the revision string. |
| `java.lang.String` | `globalInfo()`             Returns a string describing this filter |
| `boolean` | `input(Instance instance)`             Input an instance for filtering. |
| `java.lang.String` | `invertSelectionTipText()`             Returns the tip text for this property |
| `boolean` | `isOutputFormatDefined()`             Returns whether the output format is ready to be collected |
| `java.util.Enumeration` | `listOptions()`             Returns an enumeration describing the available options. |
| `static void` | `main(java.lang.String[] argv)`             Main method for testing this class. |
| `int` | `numPendingOutput()`             Returns the number of instances pending output |
| `Instance` | `output()`             Output an instance after filtering and remove from the output queue. |
| `Instance` | `outputPeek()`             Output an instance after filtering but do not remove from the output queue. |
| `void` | `setAttributeType(SelectedTag type)`             Sets the attribute type to be deleted by the filter. |
| `boolean` | `setInputFormat(Instances instanceInfo)`             Sets the format of the input instances. |
| `void` | `setInvertSelection(boolean invert)`             Set whether selected columns should be removed or kept. |
| `void` | `setOptions(java.lang.String[] options)`             Parses a given list of options. |

**Methods inherited from class weka.filters.Filter**

`batchFilterFile, filterFile, getCapabilities, getOutputFormat, isFirstBatchDone, isNewBatch, makeCopies, makeCopy, toString, useFilter, wekaStaticWrapper`

**Methods inherited from class java.lang.Object**

`equals, getClass, hashCode, notify, notifyAll, wait, wait, wait`

**Field Detail**

### TAGS\_ATTRIBUTETYPE

```
public static final Tag[] TAGS_ATTRIBUTETYPE
```

:   Tag allowing selection of attribute type to delete


**Constructor Detail**

### RemoveType

```
public RemoveType()
```


**Method Detail**

### getCapabilities

```
public Capabilities getCapabilities()
```

:   Returns the Capabilities of this filter.

    :   **Specified by:**: `getCapabilities` in interface `CapabilitiesHandler` **Overrides:**: `getCapabilities` in class `Filter`
    :   **Returns:**: the capabilities of this object **See Also:**: `Capabilities`

---


### setInputFormat

```
public boolean setInputFormat(Instances instanceInfo)
                       throws java.lang.Exception
```

:   Sets the format of the input instances.

    :   **Overrides:**: `setInputFormat` in class `Filter`
    :   **Parameters:**: `instanceInfo` - an Instances object containing the input instance structure (any instances contained in the object are ignored - only the structure is required). **Returns:**: true if the outputFormat may be collected immediately **Throws:**: `java.lang.Exception` - if the inputFormat can't be set successfully

---


### input

```
public boolean input(Instance instance)
```

:   Input an instance for filtering.

    :   **Overrides:**: `input` in class `Filter`
    :   **Parameters:**: `instance` - the input instance **Returns:**: true if the filtered instance may now be collected with output().

---


### batchFinished

```
public boolean batchFinished()
                      throws java.lang.Exception
```

:   Signify that this batch of input to the filter is finished.

    :   **Overrides:**: `batchFinished` in class `Filter`
    :   **Returns:**: true if there are instances pending output **Throws:**: `java.lang.Exception` - if something goes wrong

---


### output

```
public Instance output()
```

:   Output an instance after filtering and remove from the output queue.

    :   **Overrides:**: `output` in class `Filter`
    :   **Returns:**: the instance that has most recently been filtered (or null if the queue is empty).

---


### outputPeek

```
public Instance outputPeek()
```

:   Output an instance after filtering but do not remove from the
    output queue.

    :   **Overrides:**: `outputPeek` in class `Filter`
    :   **Returns:**: the instance that has most recently been filtered (or null if the queue is empty).

---


### numPendingOutput

```
public int numPendingOutput()
```

:   Returns the number of instances pending output

    :   **Overrides:**: `numPendingOutput` in class `Filter`
    :   **Returns:**: the number of instances pending output

---


### isOutputFormatDefined

```
public boolean isOutputFormatDefined()
```

:   Returns whether the output format is ready to be collected

    :   **Overrides:**: `isOutputFormatDefined` in class `Filter`
    :   **Returns:**: true if the output format is set

---


### listOptions

```
public java.util.Enumeration listOptions()
```

:   Returns an enumeration describing the available options.

    :   **Specified by:**: `listOptions` in interface `OptionHandler`
    :   **Returns:**: an enumeration of all the available options.

---


### setOptions

```
public void setOptions(java.lang.String[] options)
                throws java.lang.Exception
```

:   Parses a given list of options.


    Valid options are:

    ```
     -T <nominal|numeric|string|date|relational>
      Attribute type to delete. Valid options are "nominal", 
      "numeric", "string", "date" and "relational".
      (default "string")
    ```

    ```
     -V
      Invert matching sense (i.e. only keep specified columns)
    ```

    :   **Specified by:**: `setOptions` in interface `OptionHandler`
    :   **Parameters:**: `options` - the list of options as an array of strings **Throws:**: `java.lang.Exception` - if an option is not supported

---


### getOptions

```
public java.lang.String[] getOptions()
```

:   Gets the current settings of the filter.

    :   **Specified by:**: `getOptions` in interface `OptionHandler`
    :   **Returns:**: an array of strings suitable for passing to setOptions

---


### globalInfo

```
public java.lang.String globalInfo()
```

:   Returns a string describing this filter

    :   **Returns:**: a description of the filter suitable for displaying in the explorer/experimenter gui

---


### attributeTypeTipText

```
public java.lang.String attributeTypeTipText()
```

:   Returns the tip text for this property

    :   **Returns:**: tip text for this property suitable for displaying in the explorer/experimenter gui

---


### setAttributeType

```
public void setAttributeType(SelectedTag type)
```

:   Sets the attribute type to be deleted by the filter.

    :   **Parameters:**: `type` - a TAGS\_ATTRIBUTETYPE of the new type the filter should delete

---


### getAttributeType

```
public SelectedTag getAttributeType()
```

:   Gets the attribute type to be deleted by the filter.

    :   **Returns:**: the attribute type as a selected tag TAGS\_ATTRIBUTETYPE

---


### invertSelectionTipText

```
public java.lang.String invertSelectionTipText()
```

:   Returns the tip text for this property

    :   **Returns:**: tip text for this property suitable for displaying in the explorer/experimenter gui

---


### getInvertSelection

```
public boolean getInvertSelection()
```

:   Get whether the supplied columns are to be removed or kept

    :   **Returns:**: true if the supplied columns will be kept

---


### setInvertSelection

```
public void setInvertSelection(boolean invert)
```

:   Set whether selected columns should be removed or kept. If true the
    selected columns are kept and unselected columns are deleted. If false
    selected columns are deleted and unselected columns are kept.

    :   **Parameters:**: `invert` - the new invert setting

---


### getRevision

```
public java.lang.String getRevision()
```

:   Returns the revision string.

    :   **Specified by:**: `getRevision` in interface `RevisionHandler` **Overrides:**: `getRevision` in class `Filter`
    :   **Returns:**: the revision

---


### main

```
public static void main(java.lang.String[] argv)
```

:   Main method for testing this class.

    :   **Parameters:**: `argv` - should contain arguments to the filter: use -h for help


---


|  |  |  |
| --- | --- | --- |
| **Overview** \| **Package** \| **Class** \| **Tree** \| **Deprecated** \| **Index** \| **Help** \| **Weka's home** | |  |
| **PREV CLASS**   **NEXT CLASS** | **FRAMES**    **NO FRAMES**     **All Classes** |  |
| SUMMARY: NESTED \| FIELD \| CONSTR \| METHOD | DETAIL: FIELD \| CONSTR \| METHOD |  |


---
